# A genome-wide association study identifies candidate genes for sleep disturbances in depressed individuals

**DOI:** 10.1186/s40246-024-00609-5

**Published:** 2024-05-22

**Authors:** Xuena Yang, Bolun Cheng, Shiqiang Cheng, Li Liu, Chuyu Pan, Peilin Meng, Chun’e Li, Yujing Chen, Jingxi Zhang, Huijie Zhang, Zhen Zhang, Yan Wen, Yumeng Jia, Huan Liu, Feng Zhang

**Affiliations:** 1 Key Laboratory of Trace Elements and Endemic Diseases of National Health and Family Planning Commission, School of Public Health, Health Science Center, Xi’an Jiaotong University, Xi’an, China; 2 Department of Psychiatry, The First Affiliated Hospital of Xi’an Jiaotong University, Xi’an, China

**Keywords:** Sleep disorders, Depression, Comorbidity, Genome-wide association study

## Abstract

**Objective:**

This study aimed to identify candidate loci and genes related to sleep disturbances in depressed individuals and clarify the co-occurrence of sleep disturbances and depression from the genetic perspective.

**Methods:**

The study subjects (including 58,256 self-reported depressed individuals and 6,576 participants with PHQ-9 score ≥ 10, respectively) were collected from the UK Biobank, which were determined based on the Patient Health Questionnaire (PHQ-9) and self-reported depression status, respectively. Sleep related traits included chronotype, insomnia, snoring and daytime dozing. Genome-wide association studies (GWASs) of sleep related traits in depressed individuals were conducted by PLINK 2.0 adjusting age, sex, Townsend deprivation index and 10 principal components as covariates. The CAUSALdb database was used to explore the mental traits associated with the candidate genes identified by the GWAS.

**Results:**

GWAS detected 15 loci significantly associated with chronotype in the subjects with self-reported depression, such as rs12736689 at *RNASEL* (*P* = 1.00 × 10^− 09^), rs509476 at *RGS16* (*P* = 1.58 × 10^− 09^) and rs1006751 at *RFX4* (*P* = 1.54 × 10^− 08^). 9 candidate loci were identified in the subjects with PHQ-9 ≥ 10, of which 2 loci were associated with insomnia such as rs115379847 at EVC2 (*P* = 3.50 × 10^− 08^), and 7 loci were associated with daytime dozing, such as rs140876133 at *SMYD3* (*P* = 3.88 × 10^− 08^) and rs139156969 at *ROBO2* (*P* = 3.58 × 10^− 08^). Multiple identified genes, such as *RNASEL*, *RGS16*, *RFX4* and *ROBO2* were reported to be associated with chronotype, depression or cognition in previous studies.

**Conclusion:**

Our study identified several candidate genes related to sleep disturbances in depressed individuals, which provided new clues for understanding the biological mechanism underlying the co-occurrence of depression and sleep disorders.

**Supplementary Information:**

The online version contains supplementary material available at 10.1186/s40246-024-00609-5.

## Introduction

Depression is a common and debilitating mental illness characterized by persistent bad mood. It has been widely recognized as the leading cause of disability in the world, with an estimated 300 million people being affected by it during their lifespan [1, 2]. Sleep disturbances are a core symptoms of depression with diverse manifestations, including insomnia, daytime dozing and chronotype, which could result in poor sleep efficiency and quality [3]. In addition, the incidence of sleep disturbances rises with age, and approximately 60% of older adults complain of sleep problems, which in turn can increase the risk of depression [4, 5]. Surprisingly, sleep disorders might even affect the outcome of depression, as they are closely associated with the severity of depression, bad treatment response and even relapse of depression [4, 6]. The co-existence of sleep disturbances and depression undoubtedly bring heavy burden to the social economy and health care system.

There is convincing evidence that a bidirectional relationship exists among depression and sleep disturbances. Indeed, the frequency of sleep disturbances tends to be higher in depressed patients than that in healthy individuals, and more than 90% patients with depression complain of poor sleep quality [7]. It was estimated that 75% depressed individuals experienced insomnia, and 40% youngsters and 10% elderly people with depression suffered from hypersomnia [8]. A study reported a statistically significant correlation between depressive symptoms and sleep quality in adults with depression [9]. Moreover, insomnia with short sleep could indirectly increase the risk of suicide by affecting the development of depression [10]. Numerous meta-analyses have shown that individuals with insomnia symptoms are more likely to suffer depression than those without sleep problems, and insomnia could largely predict the onset and development of depression [11, 12]. Interestingly, Roman et al. found that sleep disturbances could gradually attenuate the sensitivity of serotonergic receptor, which is associated with the reduction of cognitive function and an increased risk of depression [13]. Although the complex bidirectional relationship between sleep disturbances and development of depression has been confirmed, the exploration of the underlying biological mechanisms of the co-occurrence of sleep disorders and depression is still limited.

A growing number of studies have indicated that genetic factor may exert important influence on the development of sleep disturbances and depression [1, 14–16]. The heritability of sleep disturbances and depression has been estimated at about 17–45% and 31–42%, respectively [15, 17]. Lane et al. identified several candidate loci and genes associated with insomnia and excessive daytime sleepiness, and replicated a locus significantly related to sleep duration by the genome-wide association study (GWAS) [16]. In addition, 15 loci were found to have genome-wide significant association with the self-reported morningness, and 7 loci mapped to the circadian genes, such as *RGS16* and *VIP* [14]. Moreover, a meta-analysis of three GWASs of depression has also indicated the polygenic trait of depression, and identified 102 variants and 269 genes that were statistically associated with depression [1]. Howard and his colleague conducted GWASs of three depression related phenotypes in the UK Biobank cohort, including broad depression, probable major depressive disorder (MDD) and International Classification of Diseases-code MDD, and they observed 17 genetic variants significantly related to depression [18]. Collectively, these studies suggested that sleep disturbances and depression have high heritability and complicated genetic architecture. It is noteworthy that research on the genetic factors related to sleep disturbances in depressed individuals and comorbid risk factors for both disorders is limited.

In the present study, GWASs of four sleep disturbance phenotypes were conducted in depressed samples from the UK Biobank. Then, the CAUSALdb database were used to replicate and validate whether the identified genes in our GWAS also showing genetic effects on sleep and other mental disorders in generally populations. Our results may reveal the underling genetic mechanism of high comorbidity of sleep disturbances and depression.

## Materials and methods

### UK Biobank cohort

The analysis data were extracted from the UK Biobank (application 46,478), a large-scale population based prospective cohort involving more than 500,000 subjects aged 40–69 years between 2006 and 2010 [19]. The demographics, lifestyle and health-related records of all participants were obtained via questionnaire, interview and physical measurements. In addition, the collected specimens were mainly used for biochemical detection and genome-wide genotyping, including blood, urine and saliva. All individuals gave informed consent. Our study was conducted under the approval of the UK Biobank and obtained the health-related information of participants involving depression and sleep status and genotyping data of each individual.

### UK Biobank phenotypes of depression and sleep

The depression was evaluated based on the Patient Health Questionnaire (PHQ-9) and self-reported depression status [20]. PHQ-9 is a reliable tool for assessing the severity of depressive disorder, with a total score of 0–27. Notably, PHQ-9 ≥ 10 represents moderate to severe depression and its sensitivity and specificity for major depression are 88% and 88%, respectively [20]. In addition, self-reported depression was defined according to the code 1286 from ID 20,002, code 3, 4 or 5 from ID 20,126 and code 11 from ID 20,544 of UK Biobank. In the present study, individuals with PHQ-9 ≥ 10 or self-reported depression were selected as depression cases. More detailed definition of depression has been described elsewhere [21].

The sleep-related phenotypes in this study included chronotype, insomnia, snoring and daytime dozing. Chronotype, commonly named circadian preference, represents a person’s tendency to sleep early or late [22]. In the UK Biobank, participants’ chronotype was assessed by the code 100,342 from ID 1180. Insomnia means a person who has trouble in initiating or maintaining sleep [11]. The insomnia (MIM code: 60,072) was evaluated by the code 100,343 from ID 1200. Moreover, snoring (MIM code: 107,650) refers to the unpleasant inspiratory sound caused by the upper airway during sleep [23]. The snoring was assessed by the code 100,345 from ID 1210. Daytime dozing (MIM code:161,400) represents a person who tends to fall drowsy or asleep during normal waking hours [24]. It was evaluated by the code 100,346 from ID 1220. The more detailed information of sleep-related phenotypes’ definition is shown in the Supplementary Materials.

### UK Biobank genotyping, imputation and quality control

The genotyping process, array design and quality control were described elsewhere in more detail [25]. Briefly, a total of 488,377 individuals were genotyped via either the UK Bileve array or the UK Biobank axiom array. The genotyping data were then imputed by the Haplotype Reference Consortium (HRC) reference panel [26] and a merged UK10K and 1000 Genomes phase 3 reference panels [27]. Quality control (QC) included removal of individuals who reported sex was out of line with genetic sex, who were genotyped but not imputed, who withdrew their consents and who were genetic relatedness based on self-reported “White British” (UK Biobank field ID: 21,000). The participants with genetic relatedness were removed using KING software in the UK Biobank [25, 28]. In the present study, we inferred 376,806 genetically unrelated participants with kinship coefficient less than 0.088 using the KING option ‘‘–unrelated’’ [28, 29].

### Genome-wide association study analysis

GWASs of the four sleep traits in depression samples (self-reported depressed participants: *N* = 58,256; subjects with PHQ-9 score ≥ 10: *N* = 6,576) were performed by the PLINK2 using generalized linear model with the imputed additive genetic effects, respectively [30]. In the association analyses, age, sex, Townsend deprivation index (TDI), and 10 principal components (PC) of population structure were used as covariates. An additional QC process was carried out to obtain high-quality SNPs. The exclusion criteria of SNPs were as follows: SNPs with call rate < 0.90, Hardy-Weinberg equilibrium exact test *P*-values < 0.001, minor allele frequency < 0.01, SNP-level missingness > 0.1 or individual-level missingness > 0.1. Loci with significant correlation signals were detected by *P* < 5.0 × 10^–8^. Manhattan plots and QQ plots of GWAS results were visualized in R (4.0.3) with CMplot (https://github.com/YinLiLin/CMplot).

### Exploring the mental traits related to identified candidate genes

CAUSALdb database was employed to explore the mental traits associated with the genes identified by our GWAS [31]. The database covers the most comprehensive GWAS summary data so far and identifies the potential causal genetic variants of traits/diseases via uniformly handling fine-mapping (http://mulinlab.org/causaldb/index.html). In addition, the results of related traits were confined to the European population in our study.

## Results

### Characteristics of study subjects

In our study, about 5,938 − 58,256 participants remained in GWAS analysis. As indicated by Table 1, the total sample of each sleep phenotype represents the sample size of each GWAS analysis. Among 58,256 self-reported depressed participants, 52,688 individuals (34,792 women, 17,896 men) completed chronotype assessment, and mean age (SD) was 55.74 (7.74) years old. Among 6,576 subjects with PHQ-9 score ≥ 10, 5,940 individuals (3,753 women, 2,187 men) completed chronotype assessment, and mean age (SD) was 53.12 (7.62) years old. More detailed characteristics of subjects are exhibited in the Table 1 and Supplementary Table 1.

**Table 1:** Characteristics of study subjects from the UK Biobank

	Phenotypes (MIM code)	Total sample	Women	Age (Mean ± SD)
Self-reported depression *N* = 58,256	Chronotype	52,688	34,792	55.74 ± 7.74
	Insomnia (60072)	58,012	37,788	55.72 ± 7.74
	Snoring (107650)	53,263	34,423	55.67 ± 7.74
	Daytime dozing (161400)	57,864	37,696	55.72 ± 7.74
PHQ-9 ≥ 10 *N* = 6,576	Chronotype	5,940	3,753	53.12 ± 7.62
	Insomnia (60072)	6,550	4,107	53.13 ± 7.67
	Snoring (107650)	5,938	3,684	53.05 ± 7.66
	Daytime dozing (161400)	6,533	4,095	53.12 ± 7.67

Notes: age was described as Mean ± standard deviation (SD); PHQ-9 is a reliable tool for evaluating the severity of depression with a total score of 0–27, and PHQ-9 ≥ 10 represents moderate to severe depression

Abbreviations: PHQ, Patient Health Questionnaire

### GWAS results of sleep traits in self-reported depressed subjects

**Figure 1:**
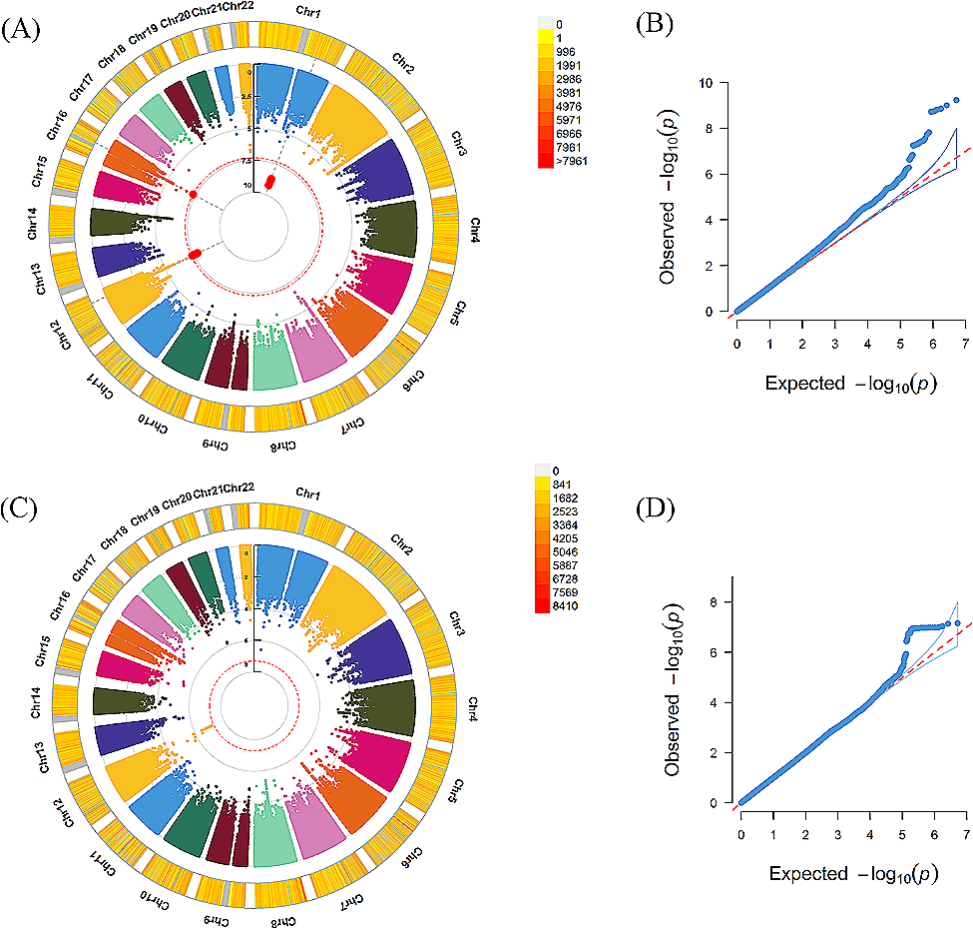
Results of genome-wide association study of chronotype and daytime dozing in self-reported depression individuals (**A**) (**C**) exhibited Manhattan plots of chronotype, daytime dozing, respectively. From the center, the first circle shows –log10p value of each variant. The second circle represents the chromosome density. Red plots indicate *P* < 5.0 × 10^–8^. (**B**) (**D**) exhibited QQ plots of chronotype, daytime dozing, respectively. A graphical representation of the deviation of the observed *p* values from the null hypothesis: the observed *p* values for each single nucleotide polymorphism (SNP) are sorted from largest to smallest and plotted against expected values from a theoretical χ^2^-distribution

**Figure 2:**
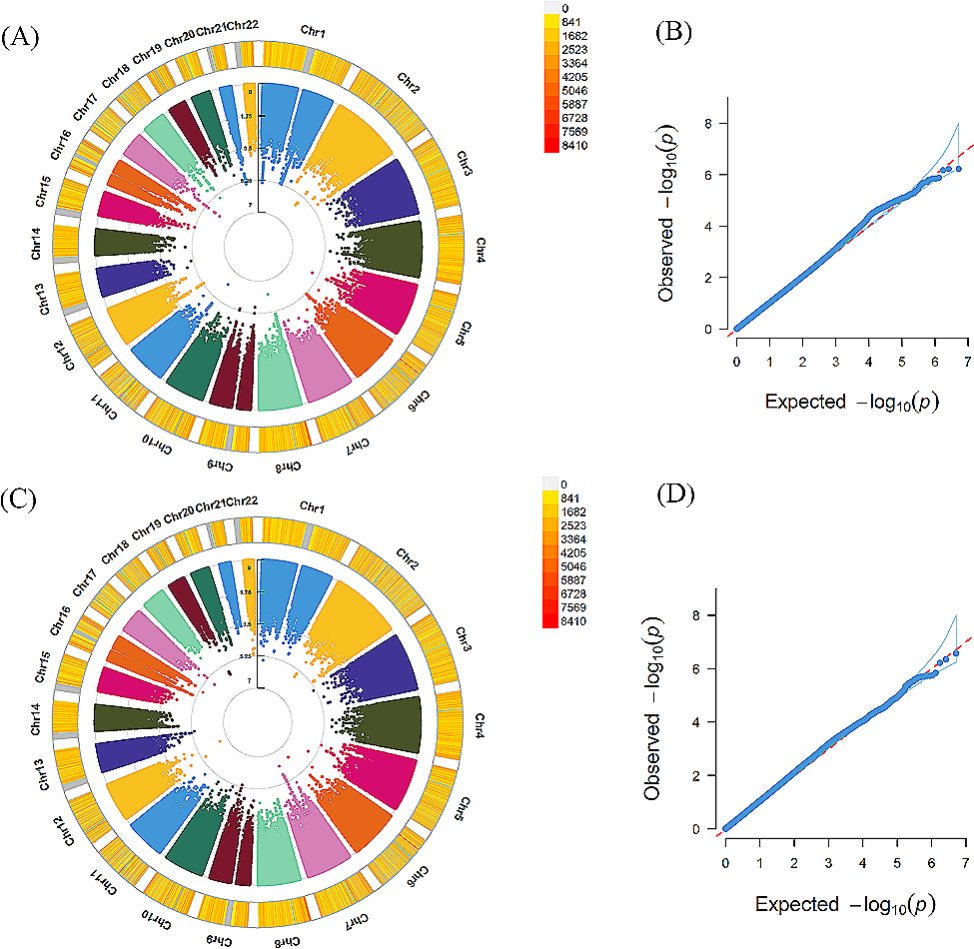
Results of genome-wide association study of insomnia and snoring in self-reported depression individuals (**A**) (**C**) exhibited Manhattan plots of insomnia and snoring, respectively. From the center, the first circle shows –log10p value of each variant. The second circle represents the chromosome density. Red plots indicate *P* < 5.0 × 10^–8^. (**B**) (**D**) exhibited QQ plots of insomnia and snoring, respectively. A graphical representation of the deviation of the observed *p* values from the null hypothesis: the observed *p* values for each single nucleotide polymorphism (SNP) are sorted from largest to smallest and plotted against expected values from a theoretical χ^2^-distribution

In the self-reported depressed individuals, GWAS detected 15 loci significantly associated with the chronotype, such as rs12736689 and rs12743617 located in *RNASEL* (MIM code: 180435, *P* = 1.00 × 10^− 09^, *P* = 1.40 × 10^− 09^, respectively), rs509476 and rs1144566 mapped to *RGS16* (MIM code: 602514, *P* = 1.58 × 10^− 09^, *P* = 1.80 × 10^− 09^, respectively). Notably, 7 of 15 loci were mapped to *RFX4* (MIM code: 603958), such as rs1006751 (*P* = 1.54 × 10^− 08^), rs4964181 (*P* = 2.73 × 10^− 08^) and rs4964472 (*P* = 3.42 × 10^− 08^). There were no significant loci correlated with insomnia or daytime dozing or snoring. GWAS results of sleep disturbances in self-reported depressed subjects are exhibited in the Table 2; Figs. 1 and 2.

**Table 2:** GWAS results of sleep disturbances in the depressed individuals meeting genome-wide significance

	Phenotypes (MIM code)	SNP	CHR	Genes (MIM code)	*P*-value
Self-reported depression	Chronotype	rs12736689	1	RNASEL (180435)	1.00 × 10^− 09^
		rs12743617	1	RNASEL (180435)	1.40 × 10^− 09^
		rs509476	1	RGS16 (602514)	1.58 × 10^− 09^
		rs1144566	1	RGS16 (602514)	1.80 × 10^− 09^
		rs694383	1	RGS16 (602514)	1.93 × 10^− 09^
		rs1006751	12	RFX4 (603958)	1.54 × 10^− 08^
		rs10778533	12	AC078929.1	1.88 × 10^− 08^
		rs4964181	12	RFX4 (603958)	2.73 × 10^− 08^
		rs4964472	12	RFX4 (603958)	3.42 × 10^− 08^
		rs7314574	12	RFX4 (603958)	3.64 × 10^− 08^
		rs2374624	12	RFX4 (603958)	3.80 × 10^− 08^
		rs919092	12	RFX4 (603958)	4.05 × 10^− 08^
		rs7299692	12	RFX4 (603958)	4.28 × 10^− 08^
		rs4784236	16	AC026462.4	4.74 × 10^− 08^
		rs4132615	16	AC026462.4	4.82 × 10^− 08^
PHQ-9 ≥ 10	Insomnia (60072)	rs576939499	1	-	2.61 × 10^− 08^
		rs115379847	4	EVC2 (607261)	3.50 × 10^− 08^
	Daytime dozing (161400)	rs140876133	1	SMYD3 (608783)	3.88 × 10^− 08^
		rs12149725	16	-	4.28 × 10^− 08^
		rs139156969	3	ROBO2 (602431)	3.58 × 10^− 08^
		rs143411328	3	ROBO2 (602431)	3.70 × 10^− 08^
		rs185361771	8	LINC01414	2.46 × 10^− 10^
		rs192804797	8	LINC01414	2.46 × 10^− 10^
		rs536718765	8	LINC01414	2.46 × 10^− 10^

Notes: The threshold of significant loci associated with sleep disorders in the depressed subjects was set to *P* < 5.0 × 10^–8^; PHQ-9 is a tool for assessing the severity of depression with a total score of 0–27

Abbreviations: CHR, chromosome; PHQ, Patient Health Questionnaire

### GWAS results of sleep traits in the subjects with PHQ-9 ≥ 10

**Figure 3:**
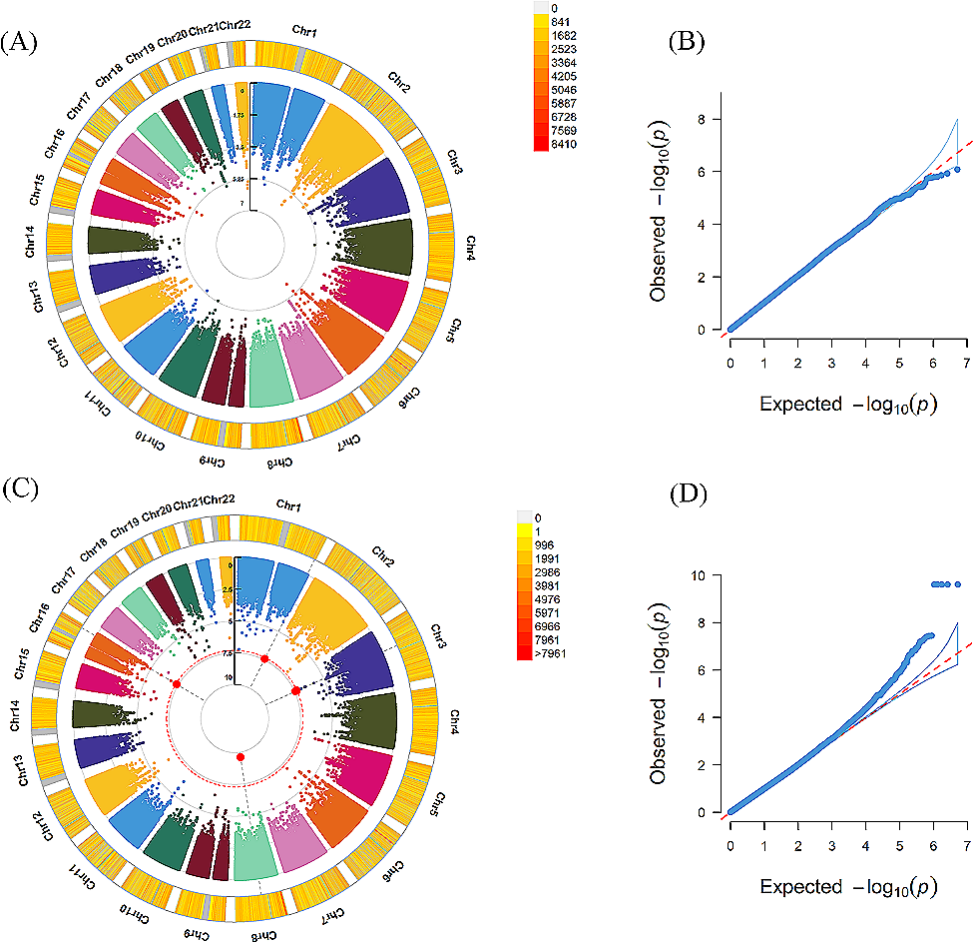
Results of genome-wide association study of chronotype and daytime dozing in the subjects with PHQ-9 ≥ 10 (A) (C) exhibited Manhattan plots of chronotype, daytime dozing, respectively. From the center, the first circle shows –log10p value of each variant. The second circle represents the chromosome density. Red plots indicate *P* < 5.0 × 10^–8^. (B) (D) exhibited QQ plots of chronotype, daytime dozing, respectively. A graphical representation of the deviation of the observed *p* values from the null hypothesis: the observed *p* values for each single nucleotide polymorphism (SNP) are sorted from largest to smallest and plotted against expected values from a theoretical χ^2^-distribution

**Figure 4:**
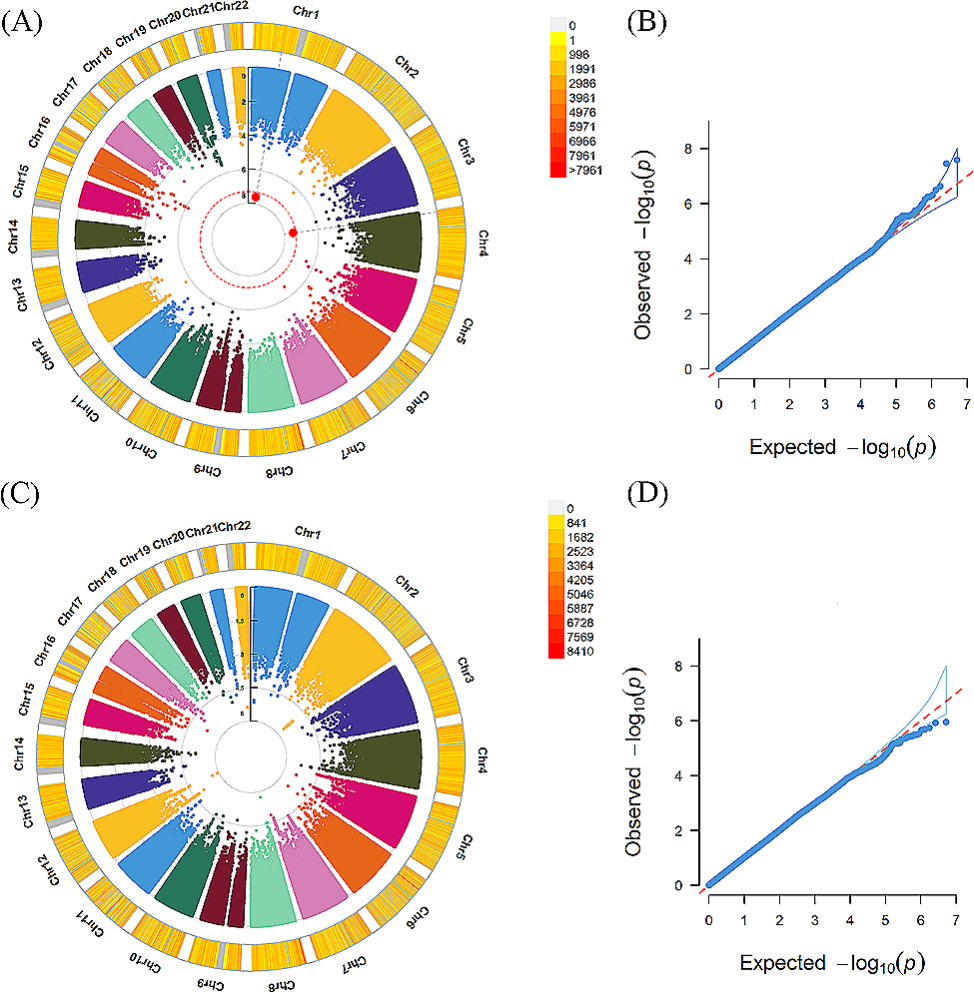
Results of genome-wide association study of insomnia and snoring in the subjects with PHQ-9 ≥ 10 (A) (C) exhibited Manhattan plots of insomnia and snoring, respectively. From the center, the first circle shows –log10p value of each variant. The second circle represents the chromosome density. Red plots indicate *P* < 5.0 × 10^–8^. (B) (D) exhibited QQ plots of insomnia and snoring, respectively. A graphical representation of the deviation of the observed *p* values from the null hypothesis: the observed *p* values for each single nucleotide polymorphism (SNP) are sorted from largest to smallest and plotted against expected values from a theoretical χ^2^-distribution

In the participants with PHQ-9 ≥ 10, GWAS identified 2 candidate loci associated with insomnia, including rs115379847 mapped to *EVC2* (MIM code: 607261, *P* = 3.50 × 10^− 08^). Additionally, 7 loci were identified for daytime dozing, such as rs140876133 located in *SMYD3* (MIM code: 608783, *P* = 3.88 × 10^− 08^), rs139156969 and rs143411328 mapped to *ROBO2* (MIM code: 602431, *P* = 3.58 × 10^− 08^*, P* = 3.70 × 10^− 08^, respectively). We did not detect significant locus associated with chronotype or snoring. The GWAS results of sleep disturbances in the subjects with PHQ-9 ≥ 10 are exhibited in the Table 2; Figs. 3 and 4

### Mental traits related to identified genes in published studies

Multiple identified genes were also reported to be associated with mental traits in the published studies, such as *RGS16* and *RNASEL* for chronotype [14, 16, 32–34], cognitive performance [35] and neuroticism [36]. *RFX4* was associated with both chronotype and depression [34]. Additionally, *ROBO2* was related to the number of depression episodes and chronotype [34]. The main results of candidate genes-related mental traits can be found in Table 3

**Table 3:** The traits associated with identified genes in the published studies

Genes	Related traits	PMID
*RGS16*/*RNASEL*	Chronotype	26835600, 27494321, 26955885, 27992416, 31427789
	Cognitive performance	30038396
	Neuroticism	29255261
	Intelligence	29942086
*RFX4*	Chronotype	31427789
	Depression	31427789
	Nap during day	31427789
*EVC2*	Depression	31427789
*SMYD3*	Depression	31427789
*ROBO2*	Number of depression episodes	31427789
	Chronotype	31427789

Notes: The candidate genes-related traits were replicated and validated in the published studies by surveying the CAUSALdb database (http://mulinlab.org/causaldb/index.html)

## Discussion

To the best of our knowledge, limited efforts have been paid to reveal the high comorbidity of sleep disturbances and depression from the genetic perspective. In the present study, using the well-established UK Biobank cohort, we explored the susceptibility genes associated with sleep disorders in depressed individuals by GWAS. We identified several candidate genes for sleep disorders in the subjects with depression, such as *RFX4*, *RGS16* and *ROBO2*.

We observed that regulatory factor X4 (*RFX4*) was significantly associated with chronotype in the self-reported depressed individuals. Furthermore, previous GWAS systematically explored the genetic architecture in 2,965 complex traits and found that RFX4 may be associated with depression and chronotype [34]. *RFX4* is widely expressed in the brain, especially in the suprachiasmatic nucleus (SCN) [37]. SCN is located in the hypothalamus directly above the optic chiasm on both sides of the third ventricle, which is also regarded as the central pacemaker point of the circadian clock, suggesting that *RFX4* may participate in the regulation of circadian rhythm patterns [38]. Interestingly, *RFX4* could be triggered by the light exposure in a subjective night-specific pattern, which is deemed as the features of several circadian genes in the SCN [38]. In addition, circadian rhythm is closely correlated with chronotype. Precisely, the chronotype differences are induced by the differences in circadian rhythm that, which regulate numerous behavioral and physiological in 24-hour cycles [39]. Thus, *RFX4* may exert essential influences on the chronotype in depressed subjects via regulating their circadian systems

Regulator of G protein signaling 16 (*RGS16*) and Ribonuclease L (*RNASEL*) were also detected to be statistically correlated with chronotype in depressed participants. *RGS16* is located in midline and intralaminar and relay nuclei of the thalamus, as well as suprachiasmatic nucleus of hypothalamus [40]. It has been widely recognized as one of circadian genes in the SCN and exerts important influence on the circadian rhythms [32]. *RGS16* could promote the synthesis of intracellular cyclic AMP in the SCN [41]. Moreover, the *RGS16* ablation was able to attenuate the circadian production of cyclic AMP, giving rise to the prolongation of circadian rhythm period, which indicated the vital relationship between chronotype and *RGS16* [41]. The clock-controlled *RGS16* precisely regulated the cyclic AMP signaling in the SCN, which could contribute the dorsomedial SCN to maintain the normal phase relationship with the ventrolateral SCN [41]. In addition, *RNASEL* is adjacent to *RGS16*, which has been shown to have significant association with chronotype [14, 42]. Kalmbach et al. explored the genetic basis of chronotype by systematically reviewing three GWASs of chronotype, and found loci statistically associated with chronotypes in *RNASEL* [42]. Moreover, study supported the association between RGS16/RNASEL and neuroticism, although they are not directly related to depression [36]. Taken together, *RGS16* and *RNASEL*, as known circadian genes, exert important impacts on the chronotype of depression individuals

Additionally, we also observed Roundabout Guidance Receptor 2 (*ROBO2*) was significantly associated with daytime dozing in depressed subjects. The associations between ROBO2 with depression and chronotype had been identified by Watanabe et al. [34]. Although the research on the relationship between *ROBO2* and daytime dozing is limited, the association of *ROBO2* and the development of dopaminergic neurons has been extensively investigated [43, 44]. *ROBO2* is broadly expressed in the developing nervous system, especially in the differentiating striatum [45]. As the essential neurotransmitter in central nervous system (CNS), dopamine exerts important influences in cognitive, sleep, mood and other neuronal functions [46]. There were huge errors in the midbrain dopaminergic axon pathfinding in *ROBO2* knockout mice, suggesting the critical roles of *ROBO2* in the establishment of dopaminergic pathways [43]. Moreover, Gore et al. found that *ROBO2* could regulate the inhibitory synaptic connectivity of ventral tegmental area, a major brain area that plays important roles in dopamine production [44]. Study reported that sleep loss could change the behaviors mainly mediated by dopamine, indicating the close relationship between dopaminergic system and sleep disturbances [47]. Therefore, *ROBO2* could indirectly exert critical influences on sleep disturbances by the dopamine, and may be a novel candidate gene correlated with daytime dozing in depressed individuals

Notably, the candidate genes identified in our study may partly explain the comorbidity of sleep disorders and depression from the genetic perspective. As circadian genes, *RFX4*, *RGS16* and *RNASEL* play important roles in the regulation of circadian rhythm systems [14, 38, 41]. The genetic variants at circadian genes may lead to slight changes in the biochemical feedback mechanism of the circadian clock and abnormal circadian systems [22]. Moreover, circadian rhythms play essential roles in the association between sleep disorders and depression [48]. The disruption of the circadian rhythm systems can induce sleep disturbances, which can increase vulnerability to depression [49]. In addition, *ROBO2* has been established to exert an important influence on the axon guidance across the midline during the development of the CNS [50]. It also plays vital roles in the establishment of dopaminergic pathways, production of dopamine, differentiation of serotonergic neuron and the expression of serotonin transporter [43, 44, 51]. Accumulating evidence has suggested that dopamine and serotonin, neurotransmitters of the CNS, play vital roles in multiple neuronal activities including sleep, cognition and emotion [46, 52]. The dopamine dysfunction has been described as participating in the relationship between sleep disturbances and depression [53]. Moreover, serotonin could regulate the circadian function, and the disruption of serotonergic system could disturb circadian rhythm systems and increase the risk of depression [54]. Interestingly, Novati et al. found that chronic sleep deprivation could induce the changes in serotonergic receptor system, which was involved in the pathophysiology of depression [55]. Thus, *RFX4*, *RGS16*, *RNASEL* and *ROBO2* may be the important genetic factors indirectly linked to sleep disturbances and depression via circadian rhythm or neurotransmitters.

Interestingly, we also observed EvC ciliary complex subunit 2 (*EVC2*) and SET and MYND domain containing 3 (*SMYD3*) significantly associated with sleep disturbances in depressed individuals. There was no statistical correlation between sleep disturbances with *EVC2* and *SMYD3* in ordinary population by surveying the CAUSALdb database. Our findings may partly reflect the different genetic factor between sleep disturbances in depressed patients and sleep disturbances in ordinary people. It is clear that depressed individuals are more prone to suffer from sleep disturbances [7]. As we mentioned above, dysregulation of neurotransmitters and disruption of circadian rhythm systems may be critical factors linking the sleep disturbances and depression. However, the exact biological mechanisms underlying the candidate genes connecting sleep disturbances with depression remain to be further elucidated.

It is noteworthy that there are several strengths in our study. On the one hand, our research identified the candidate loci and genes associated with sleep disturbances in depressed individuals, which could help us to better understand the shared genetic mechanisms of the two disorders from the genetic perspective. On the other hand, the trustworthy data was extracted from the well-established UK Biobank cohort. Moreover, the candidate genes-related traits were replicated and validated in the published studies, reflecting the credibility of our study results. In addition, our work still has some limitations. Given the study subjects were collected from the UK Biobank and were limited to “White British”, thus the findings could not be generalized to other people with different genetic background. The sleep-related phenotypes were evaluated by self-report, which could result in recall bias. Moreover, the latest whole exome sequencing (WES) data released by the UK Biobank includes more rare variants than the Human Reference Consortium (HRC) imputed genotype data, which may provide new clues for our future research.

In conclusion, our research identified some susceptibility genes associated with sleep disturbances in participants with depression. Our findings may provide novel clues for the genetic mechanism underlying sleep disturbances and depression

### Electronic supplementary material

Below is the link to the electronic supplementary material.

Supplementary Material 1

Supplementary Material 2

## Data Availability

The UK Biobank data are available through the UK Biobank Access Management System https://www.ukbiobank.ac.uk/. We will return the derived data fields following UK Biobank policy; in due course, they will be available through the UK Biobank Access Management System.
