# Supplementary material for: A genome-wide association study identifies candidate genes for sleep disturbances in depressed individuals
Source: Hum Genomics. 2024 May 22;18:51. doi: 10.1186/s40246-024-00609-5 (PMC11110369; doi:10.1186/s40246-024-00609-5)
Supplement: Supplementary file 1 — Supplementary Material 1 [file 40246_2024_609_MOESM1_ESM.docx]

***Definition of sleep-related phenotypes***

In the UK Biobank, chronotype was assessed by the touchscreen question "Do you consider yourself to be?" Participants who answered "definitely a 'morning' person", "more a 'morning' than 'evening' person", "more an 'evening' than a 'morning' person" or "definitely an 'evening' person" was separately coded "1", "2", "3" and "4" (UK Biobank field ID: 1180). Insomnia was evaluated by self-report with question "Do you have trouble falling asleep at night or do you wake up in the middle of the night?" Individuals who reported "never/rarely", "sometimes" or "usually" was correspondingly coded "1", "2" and "3", respectively (UK Biobank field ID: 1200). Snoring was determined by the question "Does your partner or a close relative or friend complain about your snoring?" Participants who answered "yes" or "no" was separately recorded "1" and "2" (UK Biobank field ID: 1210). Additionally, daytime dozing was also evaluated by self-report with question "How likely are you to doze off or fall asleep during the daytime when you don't mean to? (e.g. when working, reading or driving)". Individuals who reported "never/rarely", "sometimes", "often" or "all of the time" was correspondingly recorded "0", "1", "2" and "3" (UK Biobank field ID: 1220).
